# *In silico* identification of MAPK14-related lncRNAs and assessment of their expression in breast cancer samples

**DOI:** 10.1038/s41598-020-65421-2

**Published:** 2020-05-20

**Authors:** Sepideh Dashti, Zahra Taherian-Esfahani, Vahid Kholghi-Oskooei, Rezvan Noroozi, Sharam Arsang-Jang, Soudeh Ghafouri-Fard, Mohammad Taheri

**Affiliations:** 1 Department of Medical Genetics, Shahid Beheshti University of Medical Sciences, Tehran, Iran; 2 Department of Laboratory Sciences, School of Paramedical Sciences, Torbat Heydariyeh University of Medical Sciences, Torbat Heydariyeh, Iran; 3 Health Sciences Research Center, Torbat Heydariyeh University of Medical Sciences, Torbat Heydariyeh, Iran; 4 Malopolska Centre of Biotechnology of the Jagiellonian University, Kraków, Poland; 5 Department of Biostatistics and Epidemiology, Cancer Gene Therapy Research Center, Faculty of Medicine, Zanjan University of Medical Sciences, Zanjan, Iran; 6 Urogenital Stem Cell Research Center, Shahid Beheshti University of Medical Sciences, Tehran, Iran

**Keywords:** Cancer, Genetics

## Abstract

Mitogen-activated protein kinase (MAP kinase) pathways participate in regulation of several cellular processes involved in breast carcinogenesis. A number of non-coding RNAs including both microRNAs (miRNAs) and long non-coding RNAs (lncRNAs) regulate or being regulated by MAPKs. We performed an *in-silico* method for identification of MAPKs with high number of interactions with miRNAs and lncRNAs. Bioinformatics approaches revealed that MAPK14 ranked first among MAPKs. Subsequently, we identified miRNAs and lncRNAs that were predicted to be associated with MAPK14. Finally, we selected four lncRNAs with higher predicted scores (*NORAD*, *HCG11*, *ZNRD1ASP* and *TTN-AS1*) and assessed their expression in 80 breast cancer tissues and their adjacent non-cancerous tissues (ANCTs). Expressions of *HCG11* and *ZNRD1ASP* were lower in tumoral tissues compared with ANCTs (P values < 0.0001). However, expression levels of *MAPK14* and *NORAD* were not significantly different between breast cancer tissues and ANCTs. A significant association was detected between expression of *HCG11* and estrogen receptor (ER) status in a way that tumors with up-regulation of this lncRNA were mostly ER negative (P value = 0.04). Expressions of *ZNRD1ASP* and *HCG11* were associated with menopause age and breast feeding duration respectively (P values = 0.02 and 0.04 respectively). There was a trend towards association between *ZNRD1ASP* expression and patients’ age of cancer diagnosis. Finally, we detected a trend toward association between expression of *NORAD* and history of hormone replacement therapy (P value = 0.06). Expression of *MAPK14* was significantly higher in grade 1 tumors compared with grade 2 tumors (P value = 0.02). Consequently, the current study provides evidences for association between lncRNA expressions and reproductive factors or tumor features.

## Introduction

Mitogen-activated protein kinase (MAP kinase) pathway convey and intensify cellular messages participated in the cell proliferation. Consequently, this pathway determines cancer cell proliferation, malignant behavior of tumors and patients’ outcome in breast cancer^1^. Experiments have shown over-expression of MAP kinase in a significant percentage of breast tumors express compared with the adjacent benign tissues^1^. Moreover, somatic mutations in breast cancer tissues which resulted in dysregulation of MAPK pathways have induced immune escape associated with poor patients’ outcome^2^. Notably, immunotherapeutic approaches against MAPK signaling have resulted in favorable results^2^. Several long non-coding RNAs (lncRNAs) and microRNAs (miRNAs) have been shown to regulate MAPK pathway^3^. Among lncRNAs with fundamental roles in carcinogenesis are those acting as decoys for miRNAs to control transcription of coding genes by competing endogenous RNAs (ceRNAs)^4^. Some of these ceRNAs have been shown to enhance breast cancer evolution through alteration of MAPK signaling^5^ or other routes^6^. Based on the importance of MAPK signaling in breast cancer pathogenesis^1^ and availability of MAPK-targeting therapies^2^, identification of regulatory mechanisms of this pathway has practical significance. The interference between ceRNAs via common miRNAs characterizes a new level of gene regulation that participates in the evolution of human malignancies. Such interferences can be anticipated according to the intersection of miRNA-binding sites^7^.

In the present investigation, we aimed at identification of MAPK-related lncRNAs with putative ceRNA function. Through an *in silico* approach, we detected *MAPK14* as the most interacting RNA with miRNAs and lncRNAs. Consequently, we focused on this gene to identify the lncRNAs with putative interaction with it. Finally, we assessed expression of MAPK14-related lncRNAs in breast cancer samples and adjacent non-cancerous tissues (ANCTs).

## Methods

### *In silico* analyses

**Figure 1:**
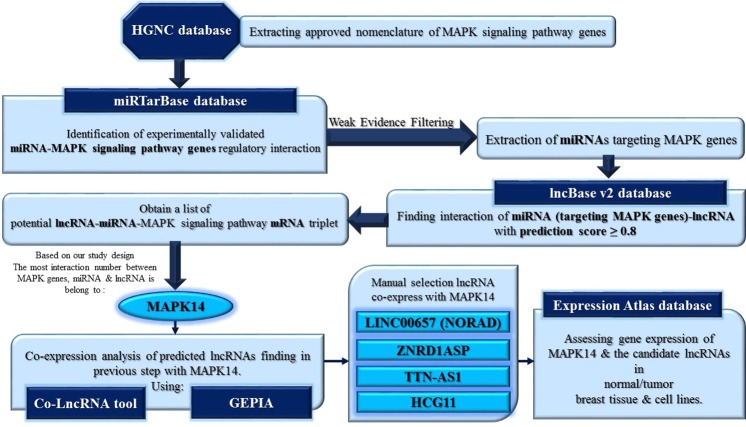
The flowchart of in silico assays to find MAPK14-associtaed lncRNAs with putative miRNA-sponging activities.

The total list of MAPK pathway genes were retrieved from HGNC database (https://www.genenames.org/data/genegroup/#!/group/652). The list of miRNAs identified in Homo sapiens was downloaded from Mirtarbase (http://mirtarbase.mbc.nctu.edu.tw/php/index.php) and miRNA-mRNA relationship was evaluated using this tool (based on the experimentally validated miRNA-mRNA relationship using Reporter assay and Western blotting techniques). miRNA-mRNA relationships with weak evidences were filtered. From the obtained list of miRNA-mRNA relationship with strong evidence, those associated with MAPK genes were selected. Subsequently, lncBase v2 (http://carolina.imis.athena-innovation.gr/diana_tools/web/index.php?r=lncbasev2%2Findex-experimental) was used for assessment of miRNA-lncRNA associations. The identified miRNAs from the previous step were assessed in lncBase v2 and the associated lncRNAs were retrieved. Scores>0.8 was used as the threshold. The miRNA-mRNA relationship was evaluated using Mirtarbase (http://mirtarbase.mbc.nctu.edu.tw/php/index.php) which is tool which reports these interactions based on the experimentally validated miRNA-mRNA relationship using Reporter assay and Western blotting techniques. The previous steps provided the list of lncRNA-miRNA-mRNA triplets to find the lncRNAs with potential sponging activities. Next, Expression Atlas^8^ data was used to identify MAPK genes with differential expression in breast cancer tissues vs. normal tissues. Expression of the previously identified lncRNAs has been assessed in Expression Atlas as well. Finally, Co-lncRNA (http://bio-bigdata.hrbmu.edu.cn/Co-LncRNA/) tool was applied to select lncRNAs which co-express with MAPK14 in breast tissues (Fig. 1).

### Patients

In the current project, we enrolled 80 female breast cancer patients aged between 36 and 60 (mean ± (SD) age: 49.59 ± 4.74). Malignant tissues and their corresponding ANCTs were obtained during surgery, promptly transferred in liquid Nitrogen to Genetic laboratory for gene expression analyses. All samples were also assessed by a pathologist to verify the diagnosis. Malignant samples included seven invasive lobular carcinomas, one papillary carcinoma, one ductal carcinoma *in situ* and 71 invasive ductal carcinomas. Patients were recruited from Farmanieh and Sina Hospitals during 2016–2018. All patients signed inform consent forms. The study protocol was approved by the Ethical Committee of Shahid Beheshti University of Medical Sciences.

### Expression analysis

RNA was extracted from all samples using the Hybrid-R 100 preps (GeneAll, Seoul, South Korea) according to the instructions. RNA samples were treated with DNAse I (Thermo SCIENTIFIC, Vilnius, Lithuania) to eliminate DNA contamination. Afterward, the RNA quantity and quality was assessed and cDNA was made from extracted RNA using Solis BioDyne kit (Estonia). Relative expressions of *MAPK14* and the associated lncRNAs were quantified in all samples using RealQ Plus Master Mix Green (AMPLICON, Odense, Denmark). *B2M* gene was used as normalizer. Quantitative real time PCR was executed in the rotor gene 6000 Corbett Real-Time PCR System in duplicate. The nucleotide sequences of primers are presented in Table 1.

**Table 1:** The nucleotide sequences of primers used in the current study.

Name	Sequence	Primer Length	PCR Product
*MAPK14-F*	AATGTGATTGGTCTGTTGG	19	156 bp
*MAPK14-R*	TTGGTAGATAAGGAACTGAAC	21	
*NORAD-F*	TGCCAATGTATGACAGAAGTAGAG	24	177 bp
*NORAD-R*	CCTTCATTCCTTCCAACTCCTC	22	
*HCG11-F*	GCAGTAAGCCGAGACCAC	18	222 bp
*HCG11-R*	GCAGTGAATAGTCAGCATACG	21	
*ZNRD1ASP-F*	TTAATTGCGAACCGTGTC	18	103 bp
*ZNRD1ASP-R*	TTGTGCTCAACCTCTCAG	18	
*TTN-AS-F*	AGTGCGAAATCCTGTCTTC	19	104 bp
*TTN-AS-R*	GATGATTCCCAGCCTTGAC	19	
*B2M-F*	AGATGAGTATGCCTGCCGTG	20	105 bp
*B2M-R*	GCGGCATCTTCAAACCTCCA	20	

### Statistical analyses

The significance of difference in mean values of transcript quantities between two paired groups was tested by the Kruschke’s Bayesian estimation method. A t student prior distribution was assumed for parameters with 4000 iteration and 2000 burn-outs. The 95% Highest density interval (HDI) was calculated based on the Bayesian approach. The P values were estimated from frequentist methods using quantile regression and mixed effects models. The ‘quantreg’, ‘ggplot2’, and ‘brms’ packages were used in R 3.5.2 environment. The association between tumor features and transcript levels of genes was evaluated using Chi-square test or Fisher exact test where appropriate using the Statistical Package for the Social Sciences (SPSS) v.18.0 (SPSS Inc., Chicago, IL). The significance of alteration between mean values of transcripts between discrete groups of patients was appraised using Tukey’s honest significance test. The correlation between transcript levels of genes was dignified using the regression model. For all statistical tests, the level of significance was set at P < 0.05.

## Results

### In silico assays

There were a total 60 MAPK genes in HGNC database. These genes were assessed by Mirtarbase and lncBase to find miRNA and lncRNA associations. Table 2 shows the potential lncRNA-miRNA-MAPK genes interaction based on our study design. As MAPK14 was found to have the greatest number of interactions with miRNAs and lncRNAs, subsequent steps were performed on this gene.

**Table 2:** The potential lncRNA-miRNA-MAPK genes interaction based on our study design.

Gene Symbol	HGNC ID	Chromosome	Gene Family Tag	Gene family description	Interaction number
MAPK14	6876	6p21.31	MAPK	Mitogen-activated protein kinases	69
RAF1	9829	3p25.2	MAP3K	Mitogen-activated protein kinase kinase kinases	64
MAPK1	6871	22q11.22	MAPK	Mitogen-activated protein kinases	61
MAP2K1	6840	15q22.31	MAP2K	Mitogen-activated protein kinase kinases	54
MAPK9	6886	5q35.3	MAPK	Mitogen-activated protein kinases	53
MAP4K4	6866	2q11.2	MAP4K	Mitogen-activated protein kinase kinase kinase kinases	21
MAP3K9	6861	14q24.2	MAP3K	Mitogen-activated protein kinase kinase kinases	21
MAP3K2	6854	2q14.3	MAP3K	Mitogen-activated protein kinase kinase kinases	20
MAP3K12	6851	12q13.13	MAP3K	Mitogen-activated protein kinase kinase kinases	19
MAP3K11	6850	11q13.1	MAP3K	Mitogen-activated protein kinase kinase kinases	16
MAP2K4	6844	17p12	MAP2K	Mitogen-activated protein kinase kinases	15
MAPK3	6877	16p11.2	MAPK	Mitogen-activated protein kinases	15
MAPK7	6880	17p11.2	MAPK	Mitogen-activated protein kinases	15
MAP3K5	6857	6q23.3	MAP3K	Mitogen-activated protein kinase kinase kinases	15
BRAF	1097	7q34	MAP3K	Mitogen-activated protein kinase kinase kinases	14
MAP3K8	6860	10p11.23	MAP3K	Mitogen-activated protein kinase kinase kinases	12
MAPK8	6881	10q11.22	MAPK	Mitogen-activated protein kinases	12
RPS6KA4	10433	11q13.1	MAPKAPK	Mitogen-activated protein kinase-activated protein kinases	11
MAP2K3	6843	17p11.2	MAP2K	Mitogen-activated protein kinase kinases	8
RPS6KA1	10430	1p36.11	MAPKAPK	Mitogen-activated protein kinase-activated protein kinases	8
MAP3K14	6853	17q21.31	MAP3K	Mitogen-activated protein kinase kinase kinases	7
MAP2K6	6846	17q24.3	MAP2K	Mitogen-activated protein kinase kinases	4
MAP3K10	6849	19q13.2	MAP3K	Mitogen-activated protein kinase kinase kinases	4
RPS6KA5	10434	14q32.11	MAPKAPK	Mitogen-activated protein kinase-activated protein kinases	3
MAP3K4	6856	6q26	MAP3K	Mitogen-activated protein kinase kinase kinases	3
RPS6KA3	10432	Xp22.12	MAPKAPK	Mitogen-activated protein kinase-activated protein kinases	3
MAPK11	6873	22q13.33	MAPK	Mitogen-activated protein kinases	2
MAPK13	6875	6p21.31	MAPK	Mitogen-activated protein kinases	2
MAP3K7	6859	6q15	MAP3K	Mitogen-activated protein kinase kinase kinases	2
MAP4K3	6865	2p22.1	MAP4K	Mitogen-activated protein kinase kinase kinase kinases	2
MAP2K7	6847	19p13.2	MAP2K	Mitogen-activated protein kinase kinases	1

We further listed miRNAs that were predicted to have associations with MAPK14 and listed the associated lncRNAs (Table 3). Co-expression analysis using GEPIA and Co-LncRNA tools revealed that *NORAD*, *HCG11*, *ZNRD1ASP* and *TTN-AS1* lncRNAs co-express with MAPK14 in breast tissues. Consequently, we selected these four lncRNAs for expression analysis.

**Table 3:** LncRNA-miRNA-MAPK14 triplet (Potential Competing Endogenous Triplet).

mRNA coding gene	miRNA	lncRNA
**MAPK14**	hsa-miR-27a-3p	C1orf132, DLX6-AS1, KCNQ1OT1, LINC00662, MIR4458HG, NEAT1, RASSF8-AS1, SNHG14, TOB1-AS1, TTN-AS1, ZNRD1-AS1
**HGNC ID:**6876	hsa-miR-17-5p	LINC00116, LINC00657 (NORAD), PWAR6, SNHG14, XIST, C1orf132, TMEM161B-AS1, HCG11
**Chromosome:**6p21.31	hsa-miR-155-5p	LINC00657 (NORAD), XIST
**Gene Family Tag:** MAPK	hsa-miR-34a-5p	EMX2OS, KCNQ1OT1, LINC00662, NEAT1, XIST
	hsa-miR-24-3p	GABPB1-AS1, LINC00662, LINC01094, NEAT1
	hsa-miR-199a-3p	KCNQ1OT1, TUG1, XIST
	hsa-miR-141-3p	DNM3OS, KCNQ1OT1
**Gene family**	hsa-miR-125b-5p	C1orf132, ERC2-IT1, GLIDR, KCNQ1OT1, KRTAP5-AS1, MEG3, SRRM2-AS1, STX16-NPEPL1
**description:** descriptionMitogen-activated protein kinases	hsa-miR-106a-5p	GABPB1-AS1, HCG11, LINC00116, NEAT1, XIST
	hsa-miR-125a-5p	C1orf132, ERC2-IT1, KCNQ1OT1, KRTAP5-AS1, MEG3, SRRM2-AS1, STX16-NPEPL1
	hsa-miR-124-3p	TTTY15, TMEM147-AS1, STXBP5-AS1, NEAT1, LINC00643, ERVK13-1, KCNQ1OT1
	hsa-miR-200a-3p	XIST, KCNQ1OT1, DNM3OS, NEAT1
	hsa-miR-214-3p	MIA-RAB4B, KCNQ1OT1, C1RL-AS1

### General data of patients

General demographic and clinical features of enrolled patients are summarized in Table 4.

**Table 4:** General demographic and clinical features of enrolled patients (SD: standard deviation).

Parameters	Values
Age (mean ± SD (range))	52.82 ± 13.41 (29–84)
Menarche age (mean ± SD (range))	13.13 ± 1.48 (10–18)
Menopause age (mean ± SD (range))	49.59 ± 4.74 (38–60)
First pregnancy age (mean ± SD (range))	21.35 ± 4.97 (14–37)
Breast feeding duration (months) (mean ± SD (range))	45.69 ± 46.08 (0–240)
Cancer stage (%)	
I	27.6
II	31.6
III	34.2
IV	6.6
Overall grade (%)	
I	18.1
II	52.8
III	29.2
Mitotic rate (%)	
I	42.4
II	43.9
III	13.6
Tumor size (%)	
<2 cm	29.7
>=2 cm, <5 cm	67.6
>=5 cm	2.7
Estrogen receptor (%)	
Positive	80
Negative	20
Progesterone receptor (%)	
Positive	75.3
Negative	24.7
Her2/neu expression (%)	
Positive	18.9
Negative	81.1
Hormone replacement therapy	
Positive	15
Negative	85

### Expression assays

**Figure 2:**
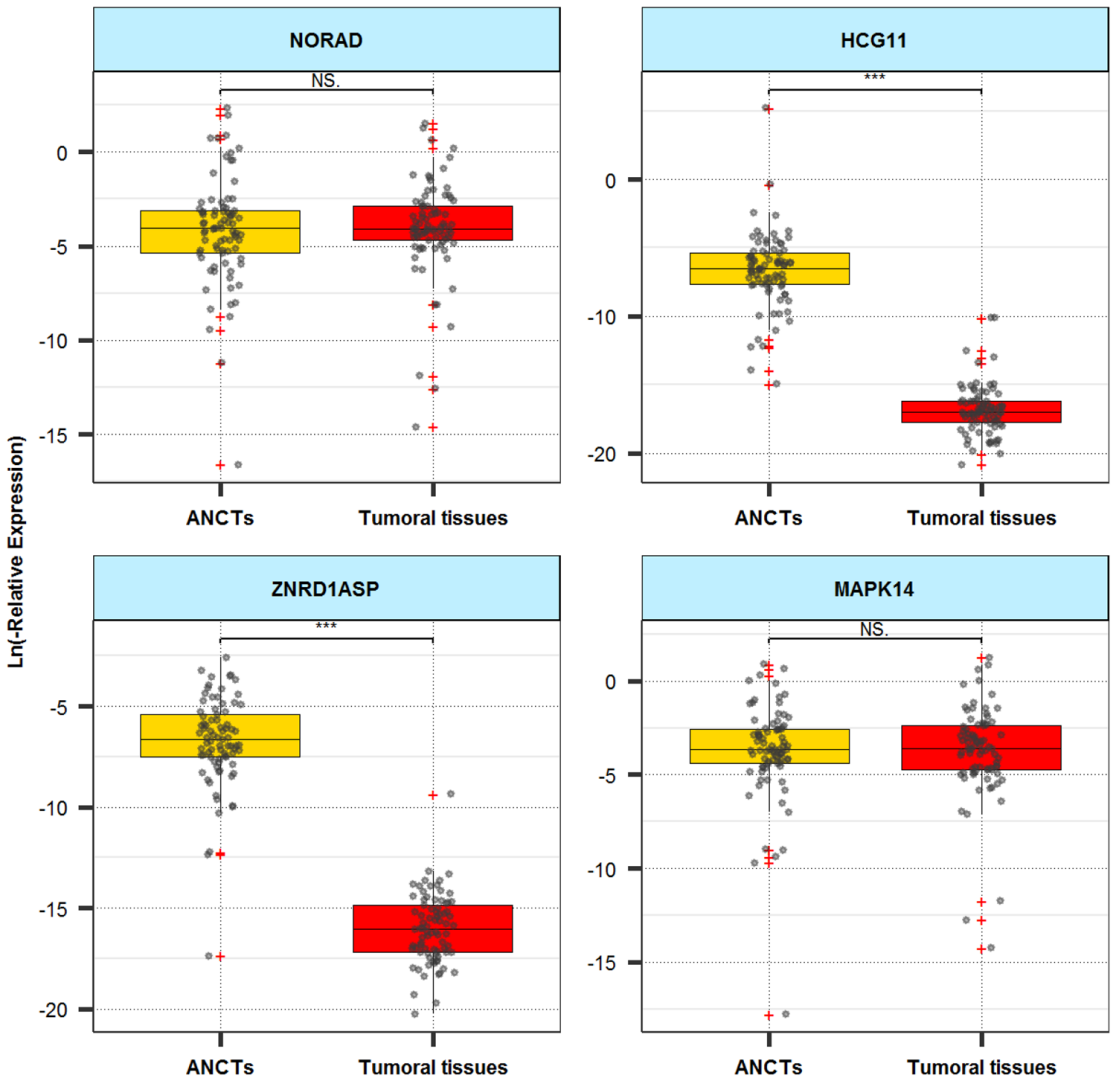
Box-Scatter plot of the expression data (qPCR) of the lncRNAs in tumor tissues vs. ANCTs.

A total of 80 breast cancer samples and 80 ANCTs were assessed. We could not detect expression of *TTN-AS1* in any of malignant or non-malignant tissues, so this gene was excluded from further steps. Expression levels of *MAPK14* and *NORAD* were not significantly different between breast cancer tissues and ANCTs. Expressions of *HCG11* and *ZNRD1ASP* were lower in tumoral tissues compared with ANCTs (P values < 0.0001). Figure 2 and Table 5 show the results of expression analysis.

**Table 5:** Bayesian t test for comparison of the relative expression of genes between two paired groups (^a^:Tumoral tissues-ANCTs; ^b^:computed from frequentist method; ^c^:95% Highest Density Interval).

Gene	Posterior mean		Relative Expression difference ^a^	Standard deviation	Effect Size	P-value^b^	95% HDI^c^
	Tumoral tissues	ANCTs					
*NORAD*	−3.869 ± 0.19	−4.104 ± 0.24	0.142	1.84	0.078	0.955	[−0.27, 0.57]
*HCG11*	−16.903 ± 0.17	−6.476 ± 0.22	−10.047	2.43	−4.211	<0.0001	[−10.68, −9.41]
*ZNRD1ASP*	−16.069 ± 0.19	−6.579 ± 0.22	−9.36	2.31	−4.09	<0.0001	[−9.91, −8.84]
*MAPK14*	−3.524 ± 0.2	−3.483 ± 0.19	−0.015	1.64	−0.01	0.783	[−0.4, 0.37]

**Figure 3:**
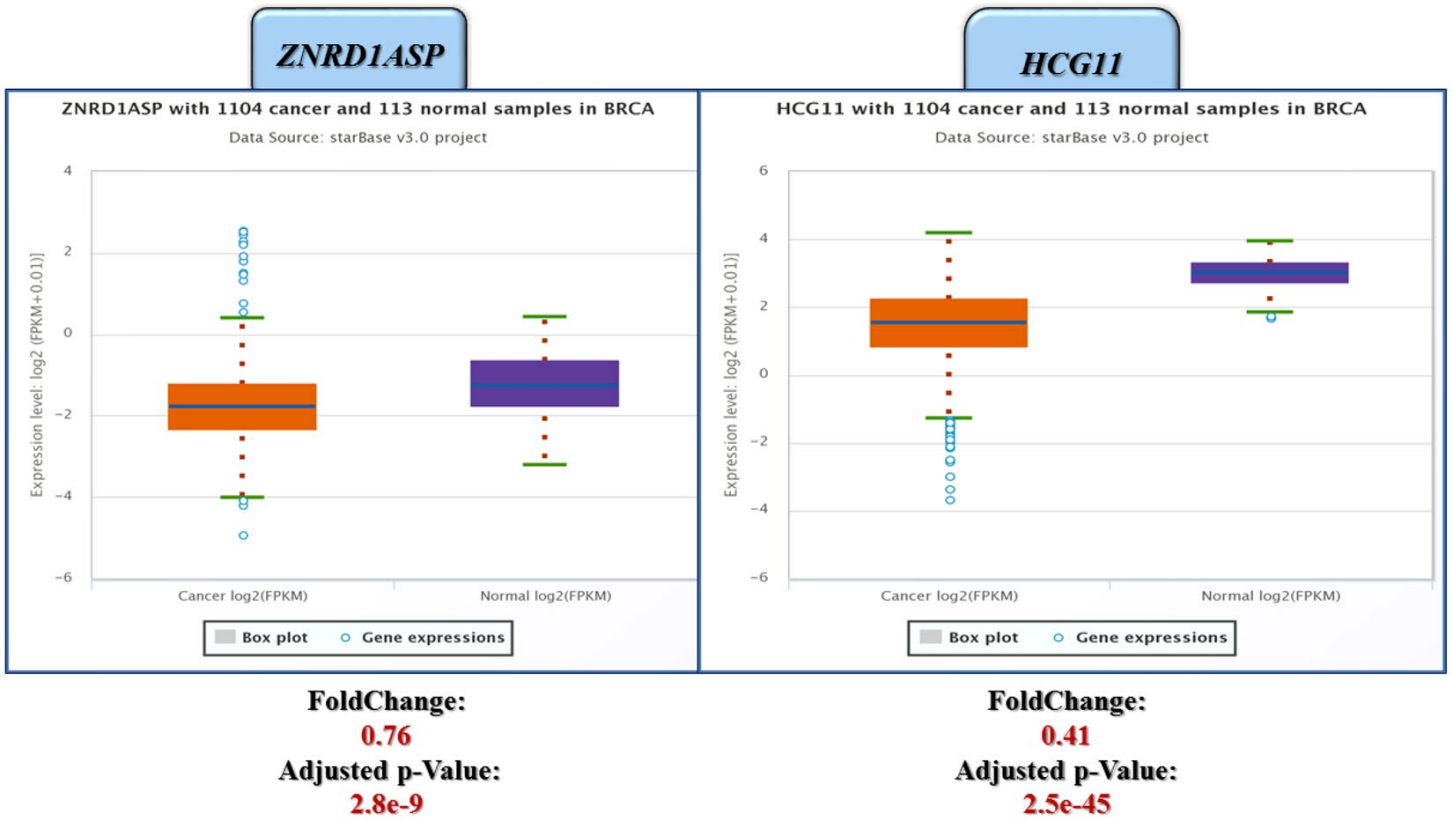
Analysis of ENCORI/Starbase v2 database showing down-regulation of *HCG11* and *ZNRD1ASP* in breast cancer tissues of TCGA project.

To further verify our results, we used ENCORI/Starbase v2 database to validate our findings in 1104 cancer and 113 normal samples from the TCGA project. Figure 3 shows that both *HCG11* and *ZNRD1ASP* are down-regulated in breast cancer tissues from TCGA database.

### Associations between expression levels of genes and patients’ features

**Figure 4:**
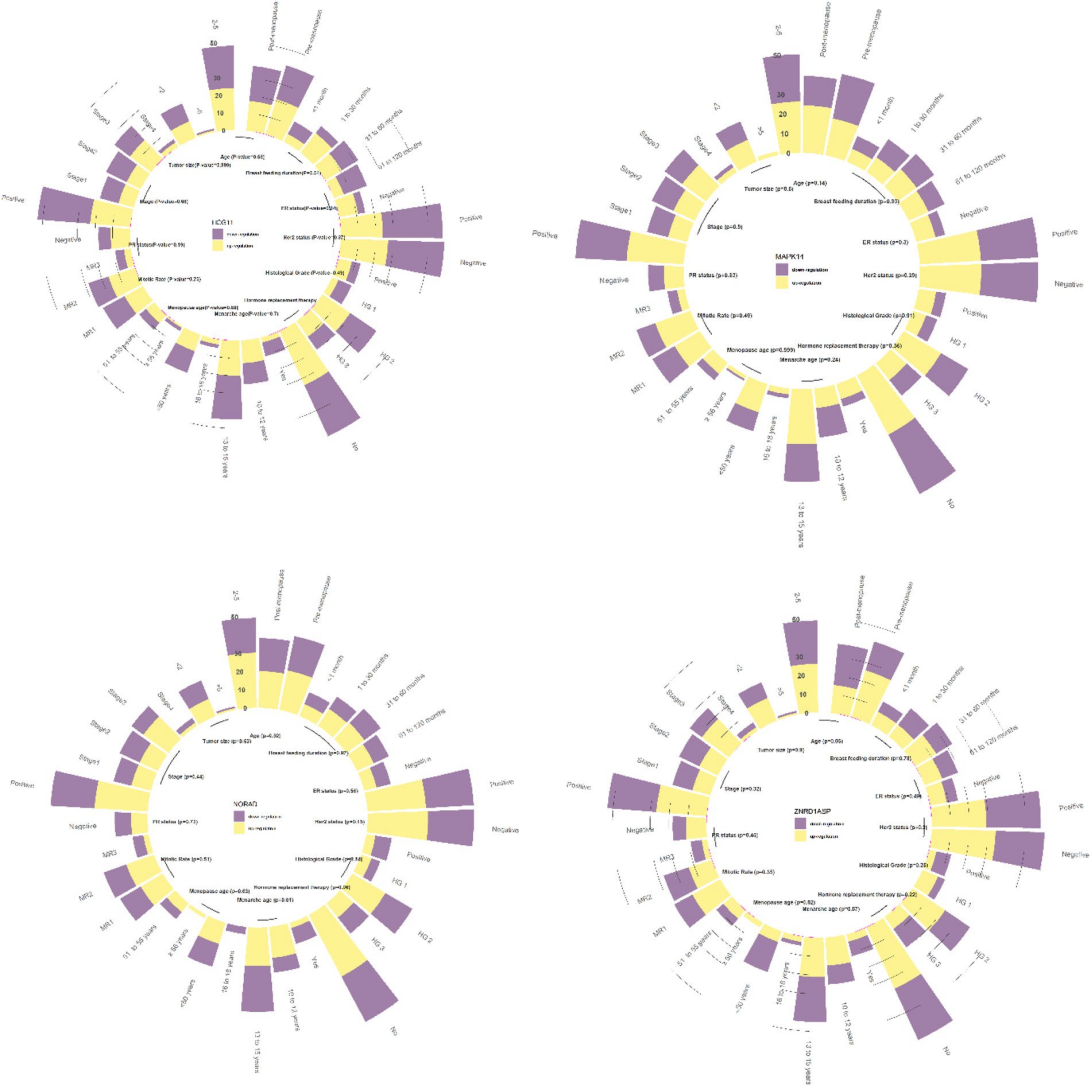
Representative results of lncRNAs down/up regulation in association with clinical parameters.

A significant association was detected between expression of *HCG11* and ER status in a way that tumors with up-regulation of this lncRNA were mostly ER negative (P value = 0.04). Besides, expressions of *ZNRD1ASP* and *HCG11* were associated with menopause age and breast feeding duration respectively (P values = 0.02 and 0.04 respectively). Moreover, there was a trend towards association between *ZNRD1ASP* expression and patients age of cancer diagnosis in a way that expression of this lncRNA tended to be up-regulated in tumor samples from pre-menopause patients compared with their paired ANCTs (P value = 0.06). Finally, we detected a trend toward association between expression of *NORAD* and history of hormone replacement therapy (P value = 0.06). Table 6 and Fig. 4 summarize the results of association analysis between expression of genes and patients’ data.

**Table 6:** The results of association analysis between expression of genes and patients’ data (Up/down regulation of genes was described according to the relative quantities of each gene in malignant tissue compared with the paired ANCT).

	*NORAD* up-regulation	*NORAD* down-regulation	P value	*HCG11* up-regulation	*HCG11* down-regulation	P value	*ZNRD1ASP* up-regulation	*ZNRD1ASP* down-regulation	P value	*MAPK14* up-regulation	*MAPK14* down-regulation	P value
Age			0.92			0.66			0.06			0.14
Pre-menopause	22 (53.7%)	19 (46.3%)		21(51.2%)	20 (48.8%)		26 (61.9%)	16 (38.1%)		19 (45.2%)	23 (54.8%)	
Post-menopause	20 (52.6%)	18 (47.4%)		17 (45.9%)	20 (54.1%)		15 (40.5%)	22 (59.5%)		24 (61.5%)	15 (38.5%)	
Stage			0.44			0.66			0.32			0.9
1	11 (52.4%)	10 (47.6%)		9 (45%)	11 (55%)		10 (47.6%)	11 (52.4%)		10 (47.6%)	11 (52.4%)	
2	11 (47.8%)	12 (52.2%)		9 (40.9%)	13 (59.1%)		11 (47.8%)	12 (52.2%)		12 (50%)	12 (50%)	
3	17 (68%)	8 (32%)		15 (57.7%)	11 (42.3%)		18 (69.2%)	8 (30.8%)		15 (57.7%)	11 (42.3%)	
4	2 (40%)	3 (60%)		3 (60%)	2 (40%)		2 (40%)	3 (60%)		3 (60%)	2 (40%)	
Histological Grade			0.84			0.49			0.25			0.91
1	6 (54.5%)	5 (45.5%)		4 (33.3%)	8 (66.7%)		9 (69.2%)	4 (30.8%)		7 (53.8%)	6 (46.2%)	
2	21 (55.3%)	17 (44.7%)		19 (51.4%)	18 (48.6%)		21 (56.8%)	16 (43.2%)		22 (57.9%)	16 (42.1%)	
3	10 (47.6%)	11 (52.4%)		11 (55%)	9 (45%)		8 (40%)	12 (60%)		11 (52.4%)	10 (47.6%)	
Mitotic Rate			0.51			0.73			0.35			0.49
1	14 (51.9%)	13 (48.1%)		12 (46.2%)	14 (53.8%)		17 (60.7%)	11 (39.3%)		15 (53.6%)	13 (46.4%)	
2	16 (55.2%)	13 (44.8%)		16 (57.1%)	12 (42.9%)		13 (48.1%)	14 (51.9%)		19 (65.5%)	10 (34.5%)	
3	3 (33.3%)	6 (66.7%)		4 (44.4%)	5 (55.6%)		3 (33.3%)	6 (66.7%)		4 (44.4%)	5 (55.6%)	
Tumor size			0.53			1			0.9			0.6
<2	10 (47.6%)	11 (52.4%)		10 (47.6%)	11 (52.4%)		13 (59.1%)	9 (40.9%)		12 (54.5%)	10 (45.5%)	
2–5	30 (61.2%)	19 (38.8%)		24 (50%)	24 (50%)		26 (53.1%)	23 (46.9%)		26 (52%)	24 (48%)	
>5	1 (50%)	1 (50%)		1 (50%)	1 (50%)		1 (50%)	1 (50%)		2 (100%)	0 (0%)	
ER status			0.56			0.04			0.49			0.3
Positive	32 (55.2%)	26 (44.8%)		24 (41.4%)	34 (58.6%)		29 (50%)	29 (50%)		31 (51.7%)	29 (48.3%)	
Negative	7 (46.7%)	8 (53.3%)		11 (73.3%)	4 (26.7%)		9 (60%)	6 (40%)		10 (66.7%)	5 (33.3%)	
PR status			0.73			0.19			0.45			0.83
Positive	29 (54.7%)	24 (45.3%)		23 (43.4%)	30 (56.6%)		27 (50.9%)	26 (49.1%)		29 (52.7%)	26 (47.3%)	
Negative	9 (50%)	9 (50%)		11 (61.1%)	7 (38.9%)		11 (61.1%)	7 (38.9%)		10 (55.6%)	8 (44.4%)	
Her2 status			0.15			0.37			0.2			0.39
Positive	5 (35.7%)	9 (64.3%)		8 (61.5%)	5 (38.5%)		4 (33.3%)	8 (66.7%)		9 (64.3%)	5 (35.7%)	
Negative	33 (56.9%)	25 (43.1%)		27 (45.8%)	32 (54.2%)		34 (56.7%)	26 (43.3%)		31 (51.7%)	29 (48.3%)	
Menarche age			0.01			0.7			0.57			0.24
10 to 12 years	17 (68%)	8 (32%)		13 (52%)	12 (48%)		15 (60%)	10 (40%)		10 (40%)	15 (60%)	
13 to 15 years	21 (45.7%)	25 (54.3%)		20 (44.4%)	25 (55.6%)		21 (46.7%)	24 (53.3%)		28 (59.6%)	19 (40.4%)	
16 to 18 years	0 (0%)	4 (100%)		3 (60%)	2 (40%)		3 (60%)	2 (40%)		3 (60%)	2 (40%)	
Menopause age			0.63			0.88			0.02			1
> 50 years	12 (48%)	13 (52%)		10 (43.5%)	13 (56.5%)		6 (26.1%)	17 (73.9%)		15 (60%)	10 (40%)	
51 to 55 years	6 (54.5%)	5 (45.5%)		6 (54.5%)	5 (45.5%)		6 (54.5%)	5 (45.5%)		7 (63.6%)	4 (36.4%)	
≥ 56 years	2 (100%)	0 (0%)		1 (33.3%)	2 (66.7%)		3 (100%)	0 (0%)		2(66.7%)	1 (33.3%)	
Breast feeding duration			0.97			0.04			0.78			0.93
0 month	7 (50%)	7 (50%)		5 (38.5%)	8 (61.5%)		8 (57.1%)	6 (42.9%)		7 (50%)	7 (50%)	
1 to 30 months	11 (57.9%)	8 (42.1%)		14 (73.7%)	5 (26.3%)		8 (42.1%)	11 (57.9%)		9 (45%)	11 (55%)	
31 to 60 months	12 (52.2%)	11 (47.8%)		11 (50%)	11 (50%)		13 (56.5%)	10 (43.5%)		12 (52.2%)	11 (47.8%)	
61 to 120 months	10 (52.6%)	9 (47.4%)		6 (30%)	14 (70%)		10 (52.6%)	9 (47.4%)		11 (55%)	9 (45%)	
Hormone replacement therapy			0.06			0.2			0.22			0.36
No	38 (57.6%)	28 (42.4%)		34 (51.5%)	32 (48.5%)		36 (54.5%)	30 (45.5%)		34 (50%)	34 (50%)	
Yes	3 (25%)	9 (75%)		3 (27.3%)	8 (72.7%)		4 (33.3%)	8 (66.7%)		8 (66.7%)	4 (33.3%)

We also compared expression of genes among distinct categories of tumor tissues (Table 7). Expression of *MAPK14* was significantly higher in grade 1 tumors compared with grade 2 tumors (P value = 0.02). No other significant difference was detected in expression of genes among distinct categories of tumors.

**Table 7:** Comparison of expression levels of genes among distinct categories of tumor tissues.

	*NORAD*	P value	*HCG11*	P value	*ZNRD1ASP*	P value	*MAPK14*	P value
ER status								
ER( + ) vs. ER(-)	0.14 (0.5) vs. 0.16 (0.5)	0.89	0.007 (0.02) vs. 0.002 (0.002)	0.52	0.008 (0.03) vs. 0.004 (0.009)	0.63	0.2 (0.6) vs. 0.1 (0.22)	0.51
PR status								
PR( + ) vs. PR(-)	0.15 (0.5) vs. 0.14 (0.46)	0.99	0.007 (0.03) vs. 0.002 (0.003)	0.45	0.008 (0.03) vs. 0.004 (0.009)	0.63	0.3 (0.63) vs. 0.1 (0.2)	0.43
HER2 status								
HER2 ( + ) vs. HER2 (-)	0.03 (0.06) vs. 0.17 (0.55)	0.34	0.003 (0.004) vs. 0.007 (0.03)	0.68	0.001 (0.001) vs. 0.008 (0.03)	0.46	0.12 (0.22) vs. 0.2 (0.6)	0.6
Tumor grade								
Grade 1 vs. 2	0.13 (0.22) vs. 0.18 (0.65)	0.94	0.004 (0.007) vs. 0.01 (0.03)	0.8	0.004 (0.004) vs. 0.01 (0.04)	0.72	0.54 (1.05) vs. 0.07 (0.15)	0.02
Grade 1 vs. 3	0.13 (0.22) vs. 0.09 (0.27)	0.98	0.004 (0.007) vs. 0.002 (0.002)	0.97	0.004 (0.004) vs. 0.002 (0.002)	0.97	0.54 (1.05) vs. 0.21 (0.54)	0.2
Grade 2 vs. 3	0.18 (0.65) vs. 0.09 (0.27)	0.79	0.01 (0.03)vs. 0.002 (0.002)	0.58	0.01 (0.04) vs. 0.002 (0.002)	0.47	0.07 (0.15) vs. 0.21 (0.54)	0.59

### Correlations between expression levels of genes

**Figure 5:**
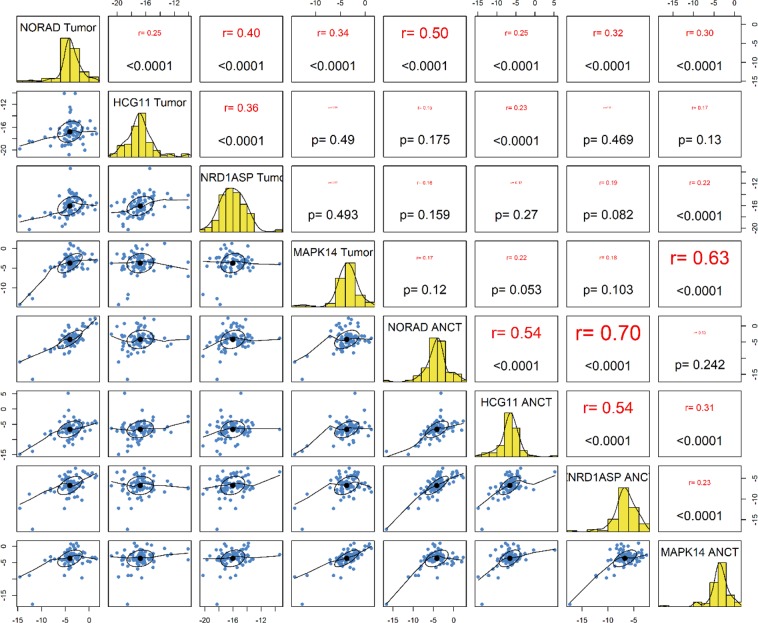
Correlation between expressions of lncRNAs in tumoral tissues and ANCTs.

A significant correlation was found between expression levels of *NORAD* and *MAPK14* in tumor tissues but not in ANCTs. There were several other pairwise correlations between expression of genes in these sets of samples (Fig. 5).

## Discussion

In the present project, we evaluated expression of *MAPK14* and four associated lncRNAs in breast cancer tissues and ANCTs. The selection of *MAPK14* was based on an *in silico* method. *In silico* studies have high chances of false positive predictions, but they are cost-benefit methods to obtain an overview of a subject before designing expensive high throughput experiments. In order to decrease chance of false positive results, we paid attention to adjusted p-values throughout the whole process and chose an FDR adjusted p-value (or q-value) lower than 0.05 as our cut off criteria.

Few previous studies have assessed the ceRNA network in breast cancer. For instance, Gao *et al*. have retrieved expression profiles of mRNAs, lncRNAs and miRNAs in invasive breast cancer from the TCGA database. They used miRcode online software to predict the interaction between lncRNAs and miRNAs. Moreover, they used TargetScan, miRDB and miRTarBase to obtain the target mRNAs of miRNAs. Assessment pf TCGA data led to identification of differential expression (DE) of 1059 lncRNAs, 86 miRNAs, and 2138 mRNAs between invasive breast cancer samples and normal samples. Subsequently, they construct an abnormal lncRNA-miRNA-mRNA ceRNA network for invasive breast cancer, consisting of 90 DElncRNAs, 18 DEmiRNAs and 26 DEmRNAs. According to the ceRNA network, they reported that the LINC00466-Hsa-mir-204- NTRK2 LINC00466-hsa-mir-204-NTRK2 axis was present in the RNAs that were correlated with patients’ outcome^9^. Their *in silico* methods were similar to the applied method in the current study. Yet, we focused on one mRNA coding gene (*MAPK14*) instead of whole transcripts. We also assessed expression of the identified genes in our cohort of patients. Le *et al*. have used the weighted gene co-expression network analysis to the available microarray mRNA and lncRNA expression data of triple negative breast cancer (TNBC) patients. They performed functional enrichment on the module that was mostly associated with Ki-67 status (Turquoise module). They also established the ceRNA network. Using this model, they have recognized correlation between two mRNAs (RAD51AP1 and TYMS) and overall survival in TNBC. Their results indicated that TNBC-specific mRNA and lncRNAs might form a complex ceRNA network which can be a putative therapeutic target for TNBC^10^. The main difference between this article and our work is inclusion of only a certain type of breast cancer in the mentioned study and assessment of the whole transcriptome.

*MAPK14* codes for α subunit of p38 MAPK. This subunit is the prototypic component of the p38 MAPK proteins that has been initially recognized as a tyrosine phosphorylated protein in triggered immune cell macrophages. In addition, MAPK14 regulates production of a number of cytokines including TNF-α^11,12^. Notably, MAPK14 has an essential role in induction of cell migration and epithelial-to-mesenchymal transition (EMT) in breast cancer cells through cooperation with TGF-β^13^. The observed similar levels of *MAPK14* between malignant tissues and ANCTs is in line with the previous finding that paracrine messages from tumor cells enhance the expression of nuclear EMT‐transcription factors in neighboring fibroblasts leading to over-expression of EMT associated genes in tumor-adjacent tissues^14^. However, some evidences point to a tumor suppressive role of MAPK14 in breast cancer. For instance, the observed enhanced MAPK14 phosphorylation in Wip1-knockout mice has been associated with lower breast tumor formation^15^. On the other hand, treatment of cancer cell lines with a certain MAPK14 inhibitor has diminished tumorigenic potential in animal models of breast cancer^16^. Notably, we detected higher levels of *MAPK14* in grade 1 tumors compared with grade 2 tumors. Taken together, one could speculate different roles for MAPK14 in each step of breast tumorigenesis. Such distinct roles have also been proposed for TGF-β (a partner of MAPK14). While in early phases of breast cancer TGF- β suppresses cell cycle transition and enhances cell apoptosis, in late phases, this cytokine is associated with augmented tumor progression, greater cell motility and malignant behavior of tumor cells^17^.

We reported lower expression of *HCG11* in tumoral tissues compared with ANCTs. We also detected a significant association between expression of *HCG11* and ER status in a way that tumors with up-regulation of this lncRNA were mostly ER negative. Liu *et al*. have previously shown associations between up-regulation of *HCG11* and poor breast cancer outcome. However, they did not report total expression changes between tumoral and non-tumoral tissues. Besides, they reported association between expression of this lncRNA and ER status^18^. Consistent with our results, this lncRNA has been previously shown to be down-regulated in prostate cancer cells and tissues^19^. Forced overexpression of *HCG11* in prostate cancer cells has suppressed cell proliferation, invasion and migration, while enhanced cell apoptosis by regulating miR‐543 expression. Besides, this lncRNA suppresses PI3K/AKT signaling pathway to inhibit progression of prostate cancer^20^. miR-543 has an inhibitory role on cell proliferation and cell cycle transition in breast cancer through modulation of ERK/MAPK^21^. Thus, the functional role of *HCG11* in breast cancer might be mediated through this miRNA.

Moreover, in line with our observation, Zhang *et al*. have demonstrated *HCG11* as an androgen-responsive lncRNA^19^. Moreover, through assessment of NONCODE data, they have detected over-expression of this lncRNA in endocrine-associated tissues such as ovary, breast and prostate, signifying its role in control of tumor evolution in these tissues^19^. Consistent with the proposed role for this lncRNA in endocrine-associated functions, we detected associations between its expression and breast feeding duration. Notably, the ceRNA network depicted by *in silico* assessments has shown participation of *HCG11* in developmental processes, differentiation, gene expression and angiogenesis^19^. Thus, down-regulation of this lncRNA in tumoral tissues might be associated with decreased differentiation state or increased angiogenic potential.

Expression of *ZNRD1ASP* was lower in tumoral tissues compared with ANCTs. Besides, expression of *ZNRD1ASP* was associated with menopause age. Moreover, there was a trend towards association between *ZNRD1ASP* expression and patients’ age of cancer diagnosis in a way that expression of this lncRNA tended to be up-regulated in tumor samples from pre-menopause patients compared with their paired ANCTs. This lncRNA is transcribed from the antisense strand of *Zinc ribbon domain containing 1* (*ZNRD1*) and negatively regulates expression of the sense transcript^22^. Previous studies have shown over-expression of *ZNRD1ASP* in lung cancer^22^. Moreover, single nucleotide polymorphisms (SNPs) within *ZNRD1ASP* modulate risk of several human cancers^22,23^.

We also reported a trend toward association between expression of *NORAD* and history of hormone replacement therapy. This lncRNA participates in the construction of a topoisomerase complex which maintains genome stability^24^. Its over-expression in breast cancer has been associated with poor patients’ survival^18^. Consistent with our data, Liu *et al*. did not detect any associations between its expression and ER, PR and HER2 status^18^.

Although *in silico* studies have shown co-expression of MAPK14 with the selected lncRNAs, we could not detect significant correlations between expression levels of lncRNAs and MAPK14 except for one case. Such lack of correlation might be explained by the high level of *MAPK14* expression and low levels of lncRNAs expressions. MAPK14 has been previously shown to be universally expressed generally at high levels^25^. So its levels of expression are expected to be very different from lncRNAs which might conceal or dilute the expected correlations. Previous studies have indicated that alterations in the ceRNA transcript levels should be adequately enormous to either conquer or decrease the miRNA effect on opposing ceRNAs^26^. Meanwhile, the observed correlations between expression levels of *NORAD* and *MAPK14* in tumor tissues in spite of lack of correlation in ANCTs implies that the interactive network between lncRNAs and MAPK14 is deregulated in the context of cancer leading to an augmented dependence or association presumably similar to what has been called as oncogene-addiction. However, further experiments are needed to verify this speculation.

## Conclusion

In brief, in the present study, we introduced an *in silico* method for identification of MAPK14-related lncRNAs with putative ceRNA role in breast cancer and assessed expression of these lncRNAs in breast cancer tissues and ANCTs. Our data supports associations between expression levels of these lncRNAs and some clinical features. Future studies are needed to elaborate the underlying mechanisms of such observations. The identified interactome comprising of MAPK14 and the 4 lncRNAs might provide new insight about the role of MAPK14 in the breast carcinogenesis and provide therapeutic targets for this cancer. As a future perspective, we can deepen the role of miRNAs in the mentioned network and assess the contribution of the selected miRNAs and their targets in the MAPK14-mediated breast carcinogenesis. Such studies would increase the insights about the regulatory mechanisms among mRNAs, lncRNAs, and miRNAs and identify promising biomarkers for breast cancer detection and treatment. Finally, this work deals with the transcriptome expression profile of MAPK14 and its associated lncRNAs. However, the effect of this interactome of MAPK14 and other interactors at the protein level were not assessed in this study which is a clear limitation of the present work.

### List of abbreviations

lncRNA (long non-coding RNA), ceRNA (competing endogenous RNA), MAP kinase (Mitogen-activated protein kinase), ANCT (adjacent non-cancerous tissue).

### Ethics approval and consent to participate

The study protocol was approved by the Ethical Committee of Shahid Beheshti University of Medical Sciences. All patients signed informed consent forms. All steps were performed according to ethical guidelines.

## Supplementary information

Supplementary Information.

Supplementary Information.

## Data Availability

The datasets used and/or analysed during the current study are available from the corresponding author on reasonable request.
