# Supplementary material for: In silico identification of MAPK14-related lncRNAs and assessment of their expression in breast cancer samples
Source: Sci Rep. 2020 May 20;10:8316. doi: 10.1038/s41598-020-65421-2 (PMC7239855; doi:10.1038/s41598-020-65421-2)
Supplement: Supplementary file 2 — Supplementary Information. [file 41598_2020_65421_MOESM2_ESM.docx]

***In silico* identification of MAPK14-related lncRNAs and assessment of their expression in breast cancer samples**

**Sepideh Dashti^1^, Zahra Taherian-Esfahani^1^, Vahid Kholghi-Oskooei^2,3^, Rezvan Noroozi^4^, Sharam Arsang-Jang^5^, Soudeh Ghafouri-Fard^1*^, Mohammad Taheri^6*^**

1. Department of Medical Genetics, Shahid Beheshti University of Medical Sciences, Tehran, Iran

2. Department of Laboratory Sciences, School of Paramedical Sciences, Torbat Heydariyeh University of Medical Sciences, Torbat Heydariyeh, Iran

3. Health Sciences Research Center, Torbat Heydariyeh University of Medical Sciences, Torbat Heydariyeh, Iran

4. Malopolska Centre of Biotechnology of the Jagiellonian University, Kraków, Poland

5. Department of Biostatistics and Epidemiology, Cancer Gene Therapy Research Center, Faculty of Medicine, Zanjan University of Medical Sciences, Zanjan, Iran

6. Urogenital Stem Cell Research Center, Shahid Beheshti University of Medical Sciences, Tehran, Iran
